# The making of the master clock

**DOI:** 10.7554/eLife.04014

**Published:** 2014-08-20

**Authors:** Ethan Buhr, Russell N Van Gelder

**Affiliations:** 1 **Ethan Buhr** is in the Department of Ophthalmology, University of Washington Medical School, Seattle, United States; 2 **Russell N Van Gelder** is in the Departments of Ophthalmology, Biological Structure, and Pathology, University of Washington Medical School, Seattle, United Statesrussvg@u.washington.edu

**Keywords:** circadian rhythms, suprachiasmatic nucleus, Lhx1, VIP, Ror-alpha, mouse

## Abstract

A genetic basis for the anatomic master circadian clock in mammals has been found.

**Related research article** Hatori M, Gill S, Mure LS, Goulding M, O'Leary DD, Panda S. 2014. Lhx1 maintains synchrony among circadian oscillator neurons of the SCN. *eLife* **3**:e03357. doi: 10.7554/eLife.03357

**Image** The suprachiasmatic nuclei (marked by arrow) are located at the base of the front half of the brain

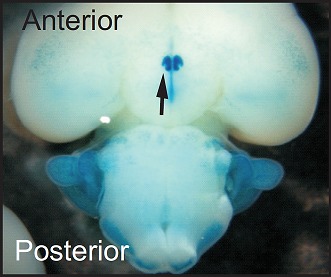

Many behaviours and physiological processes—such as hormone production and alertness—change on a roughly 24-hour cycle, called a circadian rhythm. In the early 1970s, Ronald Konopka and Seymour Benzer discovered that mutations in the *period* gene in the fruit fly *Drosophila* caused its circadian clock to change (Konopka and Benzer, 1971). Different versions of this gene resulted in flies with circadian clocks that ran fast, slow or not at all, but the flies were otherwise normal. Genetic and biochemical studies since then—looking at a range of species including *Drosophila*, *Neurospora* fungi, hamsters and mice—have revealed the existence of a relatively well conserved timekeeping mechanism.

At about the same time as the *period* mutation was discovered, a similarly remarkable finding revealed the neural basis of circadian rhythms in mammals. Applying specific lesions to a pair of structures in the brain called the suprachiasmatic nuclei (SCN) in mice resulted in the loss of all circadian rhythms, including activity, feeding and body temperature (Moore and Eichler, 1972; Stephan and Zucker, 1972). This established the SCN as the ‘master circadian pacemaker’, and remains one of the best examples of a brain nucleus having a single dedicated function. Now, in *eLife*, Satchidananda Panda and colleagues at the Salk Institute for Biological Studies—including Megumi Hatori and Shubhroz Gill as joint first authors—identify a gene that appears to give the SCN some of its circadian properties.

The question of what makes the SCN unique—particularly in terms of molecular genetic mechanisms—has not been easy to address. The clock of the SCN has characteristic responses to light exposure at different times of the day. It also produces a remarkably robust and stable oscillation, which can continue with the same period and phase in cells cultured in the laboratory for many months. It is clear that the individual SCN neurons can act as pacemakers; when dispersed in culture they independently continue to keep time (Welsh et al., 1995). However, the unique circadian properties of the SCN appear to emerge from all the cells that make up the SCN as a whole. These properties depend on the SCN cells communicating with each other, largely through neuropeptide molecules that couple the neurons. This raises a question—are there specific genes that confer this unique communication behavior on the SCN?

Two recent papers have identified LIM homeobox 1 (*Lhx1*) as such a gene. Hatori, Gill et al. used gene expression profiling—which measures the activity of thousands of genes at once—of multiple tissues in mice exposed to different circadian conditions to identify a pool of potential candidate genes. Of particular interest were genes that are regulated by light at times when the animal's behavioral circadian phase is sensitive to light exposure, and which are also expressed preferentially in the SCN.

Light exposure changed the levels of 508 gene transcripts, and the changes were strongly dependent on the time at which the mice were exposed to light. Hatori, Gill et al. then compared the set of transcripts present in the SCN with those of 82 other tissues, including 14 neural tissues, in order to find which genes are more common in the SCN. This revealed a single gene of interest: *Lhx1*. This gene expresses a transcription factor with a circadian rhythm, and the rate of transcription of *Lhx1* in a cell reduces dramatically when mice are exposed to light during the night.

In separate work Bedont et al. also identified Lhx1 as a critical transcription factor that helps set up the strength of the SCN network during development (Bedont et al., 2014). Mice without any Lhx1 die during embryogenesis. Bedont et al. therefore used a ‘floxed’ version of *Lhx1*, which could be deleted from part of the brain called the ventral hypothalamus (which includes the SCN) early in development. In the absence of hypothalamic *Lhx1*, the SCN develops into a loosely connected group of individual oscillators, incapable of driving strong circadian patterns of behavior without a daily light signal coordinating their activity. Cells in the SCN are still able to function independently as pacemakers, but their ability to express neuropeptides is largely lost, so they struggle to communicate with each other. Only when the cells are able to express these peptides correctly do the cells coalesce to create the robust self-sustained oscillations characteristic of the SCN.

The robustness of the tightly-coupled normal SCN network presents a challenge when adjustments in the oscillation phase are required—for example, when adjusting to jetlag. Every day, there are times at which the phase of the SCN is sensitive to light exposure (typically during dusk, night, and dawn) and other times of day when the phase is unaffected by light (the middle of the day). Two neuropeptides—vasoactive intestinal polypeptide and arginine vasopressin—are thought to contribute to this effect. When levels of these peptides (or their receptors) are reduced in mice, the SCN network becomes ‘looser’ and can more rapidly adapt to large jumps in the phase of the clock in response to altered lighting (Aton et al., 2005; An et al., 2013; Yamaguchi et al., 2013). The cost of this weakened coupling, however, is a weaker output signal to the systems under clock control, resulting in weaker circadian rhythms when in free-running (constant) conditions—for example, when in constant darkness.

Hatori, Gill et al. demonstrate that Lhx1 directly controls the production of these signalling peptides in adult mice (Figure 1). These mice also displayed erratic free-running behavior in the absence of light cues, but adapted almost immediately to new light–dark cycles that caused ‘jetlag’ in normal mice. It may be that the acute down-regulation of *Lhx1* after exposure to light causes a temporary weakening in the coupling between the neurons in the SCN by preventing them from communicating, which makes phase shifting easier.

**Figure 1.**
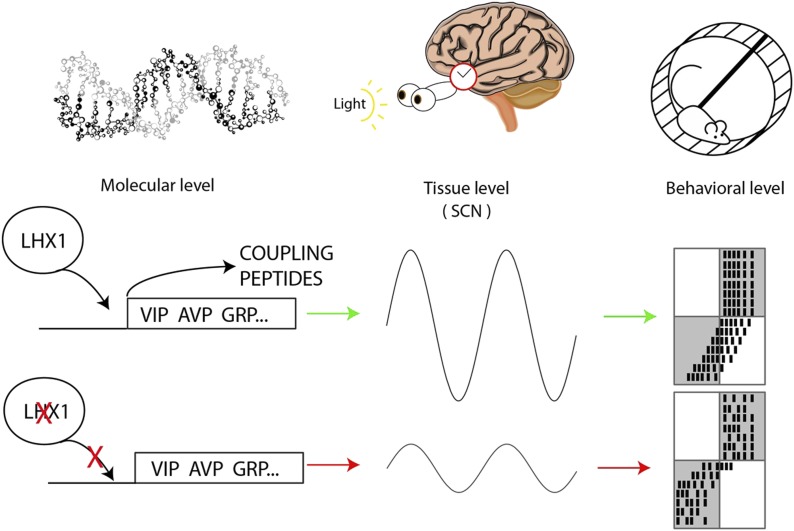
Circadian oscillations at the molecular, tissue and behavioral level. At the molecular level, the transcription factor Lhx1 controls the expression of peptides within the neurons of the SCN; when Lhx1 is not expressed, neither are the peptides. At the tissue level this peptide expression allows the coupling of the SCN neurons that produces the circadian behavior of the SCN. When Lhx1 is active, the circadian rhythms are strong; without Lhx1, the rhythms are weaker. At the behavioral level the more robust clock that results from Lhx1 activity (middle row) is slower to adapt to shifted light schedules. The white and grey boxes indicate times when lights are on (white) or off (grey). Black marks are the hypothetical daily activity of a mouse in a running wheel. The mice are normally active when it is dark. When Lhx1 is expressed, the mice find it more difficult to adapt to a sudden change in the light cycle, which results in a lot of activity when it is light. When Lhx1 is absent (bottom), the mice are better able to adjust to restrict their activity to the hours of darkness.

The combined work from these two groups reveals that Lhx1 both controls the development of the peptides the SCN neurons use to communicate, and is also a dynamic regulator of coupling strength throughout adulthood. These results highlight the continued power of genetic approaches to reveal the mechanisms of circadian rhythmicity, even as the unanswered questions move on from ‘how do the circadian oscillators inside individual cells work?’ to ‘how does the coupling of clocks within the SCN occur?’. These results also strongly suggest that Lhx1 and its downstream targets may be an attractive target for drug development, as temporarily inhibiting Lhx1 activity might significantly accelerate recovery from jet lag.
